# MALDI-TOF mass spectrometry and identification of new bacteria species in air samples from Makkah, Saudi Arabia

**DOI:** 10.1186/1756-0500-7-892

**Published:** 2014-12-09

**Authors:** Emmanouil Angelakis, Muhammad Yasir, Esam I Azhar, Anastasia Papadioti, Fehmida Bibi, Asad S Aburizaiza, Sarah Metidji, Ziad A Memish, Ahmad M Ashshi, Ahmed M Hassan, Steve Harakeh, Philippe Gautret, Didier Raoult

**Affiliations:** URMITE CNRS-IRD 198 UMR 6236, Université de la Méditerranée, Faculté de Médecine, 27 Bd Jean Moulin, 13385 Marseille, France; Special Infectious Agents Unit, King Fahd Medical Research Center, King Abdulaziz University, Jeddah, Saudi Arabia; Department of Medical Laboratory Technology, Faculty of Applied Medical Sciences, King Abdulaziz University, Jeddah, Saudi Arabia; Enviromental Science Department, Faculty of Metrology, Environmental Science and Arid Land Agriculture, King Abdulaziz University, Jeddah, Saudi Arabia; Ministry of Health, Riyadh, Saudi Arabia; Department of Laboratory Medicine, Faculty of Applied Medical Science, Umm Al-Qura University, Makkah, Saudi Arabia; Infectious Diseases and Tropical Medicine Unit - North Hospital, Chemin des Bourrelys - 13915, Marseille, France

**Keywords:** MALDI-TOF mass spectrometry, Hajj season, New bacteria species, Air contamination, Saudi Arabia

## Abstract

**Background:**

During the Hajj season, respiratory symptoms are very common among pilgrims. Here, we investigated the viable bacterial population in air samples collected around the slaughterhouses used during the Hajj.

**Methods and results:**

We collected air samples on three days from four different sites: slaughterhouses at Al-Kakia, Al-Meaisim and Al-Sharaia, and from a waste disposal area designated for the remnants of slaughter. Samples were cultured on blood agar plates for 48 h, and bacterial isolates were identified using MALDI-TOF MS. A dendrogram using the spectra of the unidentified bacterial species was constructed, and PCR amplification and sequencing of the 16S rRNA gene was performed for one isolate per cluster. In total, 2500 colonies appeared on the nutrient agar plates, and 244 were purified for further analysis. Good identification was obtained for 202 (83%) isolates by MALDI-TOF MS. The most common genera were *Bacillus* (n = 94, 45%) and *Staphyloccocus* (n = 55, 26%). Poor identification was obtained for 42 (17%) isolates, and their spectra clustering revealed that these isolates belonged to 10 species. Four of these were considered to be new species.

**Conclusions:**

During the Hajj, the air was contaminated by many environmental bacterial agents, and MALDI-TOF MS was successfully adapted for their rapid identification.

## Background

The Hajj (the Muslim pilgrimage to Makkah) is one of the largest annual gatherings in the world. Every year, more than 2 million pilgrims from over 160 countries travel to Makkah to perform the Hajj [1]. The gathering of so many people for a short period of time presents many public health challenges. Crowding, fatigue and the extreme climate conditions are significant factors that promote the transmission of airborne infections. During the Hajj season, respiratory symptoms are very common among pilgrims [2], and respiratory infections, led by pneumonia, are the most common cause of hospital admissions [3]. In one study, approximately 39% of all patients admitted for hospitalization had a respiratory infection [4]. Recently, an outbreak of respiratory syndrome coronavirus (MERS-CoV) infections was reported in Saudi Arabia. In addition, the bacterial agents most commonly responsible for pneumonia at the Hajj are *Klebsiella pneumoniae*, *Streptococcus pneumonia*, *Acinetobacter* sp., *Pseudomonas aeriginosa* and *Staphylococcus aureus* [5, 6]. Many animals are sacrificed by pilgrims or, more commonly, by slaughterhouse workers on behalf of pilgrims as a part of the Hajj rituals. Previously, contaminated sheep waste that had been left uncovered around slaughterhouses was associated with outbreaks of airborne disease in France [7, 8]. Q fever outbreaks have also been associated with airborne transmission from contaminated wastes [7, 8].

Outdoor airborne microorganisms can be found in many environments, but in most cases, they do not present a health hazard to exposed individuals. The monitoring of current levels of outdoor airborne bacterial agents is necessary to evaluate the potential risks to human health. Matrix-assisted laser desorption ionization time-of-flight (MALDI-TOF) mass spectrometry (MS) for the identification of bacterial isolates or biological samples is being used increasingly in microbiology laboratories [9]. MALDI-TOF MS has previously been used to determine the airborne bacterial community at a subway station in Norway [10]. Determining the overall bacterial diversity in outdoor atmospheres is essential to facilitate the rational development of public health policies. Special slaughterhouses and waste disposal areas have been constructed for this purpose in the Mina region near Makkah. Detection and measurement of airborne bacterial contaminants in these slaughterhouses and waste disposal areas is needed to assess contamination levels and to estimate the resulting exposure of occupants. The objective of this study was to investigate the viable bacterial population in air samples collected around slaughterhouses used during the Hajj season. To this end, we employed MALDI-TOF MS and PCR amplification and sequencing of the bacterial 16S rRNA gene [11] and we found that during the Hajj, the air was contaminated by many environmental bacterial agents and MALDI-TOF MS was successfully used for the identification of airborne bacterial contaminants.

## Results

After 24 hours of incubation, a total of 2,500 isolates appeared on the air sample nutrient agar plates. Totally, 244 colonies were purified by sub-culturing for further analysis. A total of 21 (8%) of these isolates were from Al-Kakia, 92 (38%) were from Al-Maisim, 55 (23%) were from Al-Sharaia and 76 (31%) were from the waste disposal area. During air sample collection on the first day, the temperature was 37°C and the relative humidity was 68%. The temperature on both the second and third day was 40°C and the relative humidity was 40%.

Among the 244 selected colonies tested by MALDI-TOF MS, good identification was obtained for 202 (83%). The most common genera were *Bacillus* (n = 94, 45%) and *Staphyloccocus* (n = 55, 26%). We identified 33 different bacterial species by MALDI-TOF MS including 9 different *Bacillus* sp., 7 *Staphyloccocus* sp., 3 *Pseudomonas* sp., 3 *Enterococcus* sp., 2 *Corynebacterium* sp. and 9 other bacteria species (Table 1). We totally detected 22 (66%) different species in the waste area, 17 (52%) in Al-Sharaia, 14 (42%) in Al-Maisim and 7 (21%) in Al-Kakia. *Bacillus pumilus* was the most commonly detected species (n = 31, 17%), followed by *Staphylococcus sciuri* (n = 25, 12%), *Bacillus cereus* (n = 20, 10%) and *Bacillus subtilis* (n = 20, 10%). *B. cereus* was the most commonly detected species in the waste area (n = 9, 14%), *B. pumilus* in Al-Kakia (n = 5, 36%) and Al-Sharaia (n = 8, 19%) and Al-Maisim (n = 10, 22%). *Staphylococcus aureus* was isolated only from the sites of Al-Sharaia and Al-Maisim. We did not find significant difference between the different species isolated from the waste area comparing to Al-Sharaia (*p* = 0.3) and Al-Maisim (*p* = 0.08). Significant less different species were isolated to Al-Kakia comparing to the waste area (*p* > 0.01) and Al-Sharaia (*p* = 0.02).

**Table 1:** **Areas tested and isolates identified by MALDI-TOF**

Bacteria species	Al-Kakia	Waste area	Al-Maisim	Al-Sharaia	Waste area	Al-Maisim	Al- Sharaia	Waste area	Al-Maisim	Al- Sharaia
	7 am-9 am	7 am-9 am	7 am-9 am	7 am-9 am	7 pm-9 pm	7 pm-9 pm	10 am-3 pm (1 ^st^day)	10 am-3 pm (2 ^nd^day)	10 am-3 pm (2 ^nd^day)	10 am-3 pm (2 ^nd^day)
*Alcaligenes faecalis*	2	3	1							2
*Arthrobacter gandavensis*			1			2				
*Bacillus amyloliquefaciens*				2	1	1				
*Bacillus cereus*	3	5	3	1	1	1		3	2	1
*Bacillus endophyticus*										1
*Bacillus infantis*	1	2								
*Bacillus licheniformis*				2						
*Bacillus mojavensis*	1					2				
*Bacillus pumilus*	5	5	7	3	2	1	2		3	3
*Bacillus subtilis*	1	1	2		2	7	2		2	3
*Bacillus vallismortis*		1					1			
*Bhargavaea cecembensis*					1					
*Brevundimonas diminuta*					1				1	
*Corynebacterium callunae*						1				
*Corynebacterium glutamicum*			2			1			1	
*Enterococcus casseliflavus*						2				1
*Enterococcus faecalis*		3		2		2		1	1	
*Enterococcus faecium*							1			
*Exiguobacterium aurantiacum*					1					1
*Kurthia gibsonii*						2				
*Lysinibacillus fusiformis*		2								
*Macrococcus caseolyticus*		2				1		2		2
*Pantoea calida*		2				1				
*Pseudomonas quianpuensis*			1			1				
*Pseudomonas stutzeri*		2			1	1				
*Pseudomonas xanthomarina*								1	1	
*Staphylococcus equorum*		2								
*Staphylococcus arlettae*					5	3	1		1	
*Staphylococcus gallinarum*		3								
*Staphylococcus aureus*			2			1	1	1		1
*Staphylococcus lentus*	1	2	4				2		2	1
*Staphylococcus nepalensis*						2				
*Staphylococcus sciuri*			5	2	5	5	3		3	2
	14	35	28	12	20	37	13	8	17	18

**Figure 1:**
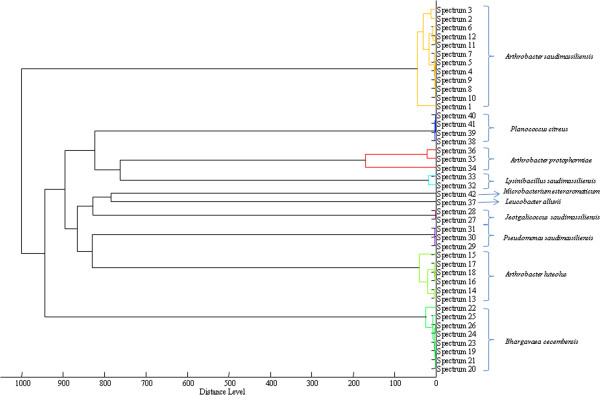
**Dendrogram of the spectra of the unidentified bacterial species by MALDI-TOF MS.** Red color, isolates selected for 16S rRNA analysis.

Poor identification was obtained for 42 isolates (17%), which were further tested by 16S rDNA amplification and sequencing. The dendrogram of spectra of the unidentified bacterial species is presented in Figure 1. This dendrogram revealed that the spectra from unidentified bacteria belonged in 10 different clusters. We considered bacteria in each cluster to be representatives of the same species. As a result, 16S rRNA sequencing was performed for only 10 isolates (one isolate per cluster). These species were identified as *Arthrobacter luteolus*, *Arthrobacter protophormiae*, *Bhargavaea cecembensis*, *Leucobacter alluvii*, *Microbacterium esteraromaticum* and *Planococcus citreus* (Table 2). In addition, we found that four isolates exhibited <98.7% nucleotide sequence similarity with *Arthrobacter nitroguajacolicus*, *Jeotgalicoccus psychrophilus*, *Pseudomonas bauzanensis* and *Lysinibacillus sphaericus*. This value was lower than the 98.7% threshold required to delineate a new species without carrying out DNA-DNA hybridization [12]. However, these isolates were considered to be new species and named *Arthrobacter saudimassiliensis* (GenBank No HG931344), *Jeotgalicoccus saudimassiliensis* (GenBank No HG931342), *Pseudomonas saudimassiliensis* (GenBank No HG931341) and *Lysinibacillus saudimassiliensis* (GenBank No HG931343). *A. saudimassiliensis* was only isolated from the waste disposal area, *J. saudimassiliensis* only from Al-Maisim whereas *P. saudimassiliensis* and *L. saudimassiliensis* existed in Al-Maisim and Al-Sharaia. The spectra for most of the unidentified bacteria were absent from the initial Brüker database, which at the time of our study contained 4,706 bacterial spectra, while our updated MALDI-TOF mass database contained 6,353. The initial Brüker database only contained one spectrum for *A. luteolus* (Table 2).

**Table 2:** **Isolates identified only by molecular biology and MALDI-TOF database**

Identification	No of isolates	MALDI-TOF database
*Arthrobacter luteolus*	6	1
*Arthrobacter saudimassiliensis*	12	0
*Bhargavaea cecembensis*	8	0
*Jeotgalicoccus saudimassiliensis*	2	0
*Leucobacter alluvii*	1	0
*Lysinibacillus saudimassiliensis*	2	0
*Microbacterium esteraromaticum*	1	0
*Planococcus citreus*	4	0
*Arthrobacter protophormiae*	3	0
*Pseudomonas saudimassiliensis*	3	0
Total	42	

## Discussion

We tested a large number of isolates from air samples, and we found that MALDI-TOF MS served as a useful tool for the identification of isolates at the species levels. Air samples collected during the Hajj season were contaminated by many environmental, but not pathogenic, bacterial species. A limitation of our study was that air samples were only tested for bacteria and not for the presence of airborne fungi, viruses, and toxins. In addition, the isolation was based on only one culture condition, a limitation that possibly decreased diversity of the isolates. Finally we did not calculate the number of isolates firstly appeared on the nutrient agars to determined which site was the most contaminated.

In our laboratory, MALDI-TOF MS is routinely used to identify bacterial species and subspecies [13]. Our findings are reliable because in cases of a bad MALDI-TOF MS identification, amplification and sequencing of the 16S rRNA gene was performed. In our laboratory, we identify approximately 32,430 isolates by MALDI-TOF MS annually [13]. Moreover we previously used MALDI-TOF MS to directly identify 233 out of 349 bacterial species from 36,500 colonies cultured from 4 stool samples [11]. For the remaining 116 unidentified species of bacteria in the same study, 16S rRNA gene sequencing was necessary [11]. However, the construction of a dendrogram from spectra of unidentified bacteria decreased the need for 16S rRNA gene sequencing. Here, because we tested environmental isolates, the MALDI-TOF database did not contain their spectra, and only 83% of the airborne isolates were correctly identified. Moreover, the presence of a low number of spectra in the database for some species did not allow MALDI-TOF to identify bacteria in the groups with biodiversity within species. In contrast, in a previous study on patient samples, MALDI-TOF MS was much more effective and identified 95% of the bacterial isolates [14].

The appropriate interpretation of air sampling results remains a challenge [15]. Previous studies have revealed that numerous bacteria and fungi are present in air [16, 17]. However, the diversity of the air microbial community is generally underestimated because most environmental microbes are resistant to culture [18]. It has been estimated that only 0.08% of the microscopically visible motile cells from outdoor air can be readily cultured [19]. A recent metagenomic study found that the most abundant airborne microbes in Singapore included several species of *Brevundimonas* [18]. Based on one small hospital survey, *Mycobacterium tuberculosis* and Gram-negative bacteria were the common causes of pneumonia during the 1994 Hajj season [20]. In addition, cases of pneumonia were also caused by *Streptococcus pneumoniae*, *Legionella pneumophilia*, and *Mycoplasma pneumoniae* [20]. In 1998, *Haemophilus influenzae*, *Klebsiella pneumoniae* and *S. pneumoniae* were found to be the most common agents of pneumonia in pilgrims [5]. In a large survey conducted of 15 intensive care units during the Hajj in 2009 and 2010, *Acinetobacter* sp., *Klebsiella* sp., *Pseudomonas aeruginosa*, *Staphylococcus aureus* and *Streptococcus pneumonia* were among the most common pathogens responsible for pneumonia [6]. Finally, during the 2005 Hajj season, the majority of pneumonia cases were caused by *Candida albicans* and *Pseudomonas aeruginosa,* followed by *L. pneumophila* and *K. pneumonia* [21]. However, the results of this survey are questionable and may have been confounded by laboratory contamination. None of these agents were found in the air samples we tested. The absence of *L. pneumophila*, *M. tuberculosis* and *H. influenzae* can be explained by the fact that we used nutrient agar for the isolation of bacteria. However, the other bacterial species associated with pneumonia in pilgrims can be isolated on blood agar.

Two surveys addressing microbiological contamination of air were conducted in the areas of Jeddah and Mecca. In the Jeddah survey, *Aspergilus fumigates* and *Aspergilus niger* were the most common fungal types isolated from air samples [22]. In the Makkah survey, airborne microbial contamination was collected from the holy mosque. Gram-positive bacteria constituted 90-100% of the total bacterial isolates. *Staphylococcus* sp. and *Bacillus* sp. were the dominant Gram-positive bacteria, which corroborates our results. Steptococci and *Pseumomonas* sp. were also isolated, with variations depending on the site of collection. *A. fumiggatus*, *A. niger*, *A. flavus* and *Fusarium* sp. were the most common fungal types isolated [23].

## Conclusions

In conclusion, during the Hajj, the air was contaminated by many environmental bacterial agents, most of which were environmental bacteria. Respiratory tract infections during Hajj continue to exert a burden on pilgrims [2]. Early identification of pathogenic bacterial clusters could lead to faster mitigation of outbreaks and a better understanding of disease etiology. Therefore, we believe that a systematic determination of the bacterial contaminants in crowded outdoor environments is essential. MALDI-TOF MS has been successfully adapted for the identification of microorganisms in clinical microbiology laboratories and in this study was successfully used for the identification of airborne bacterial contaminants.

## Methods

### Sample collection

**Figure 2:**
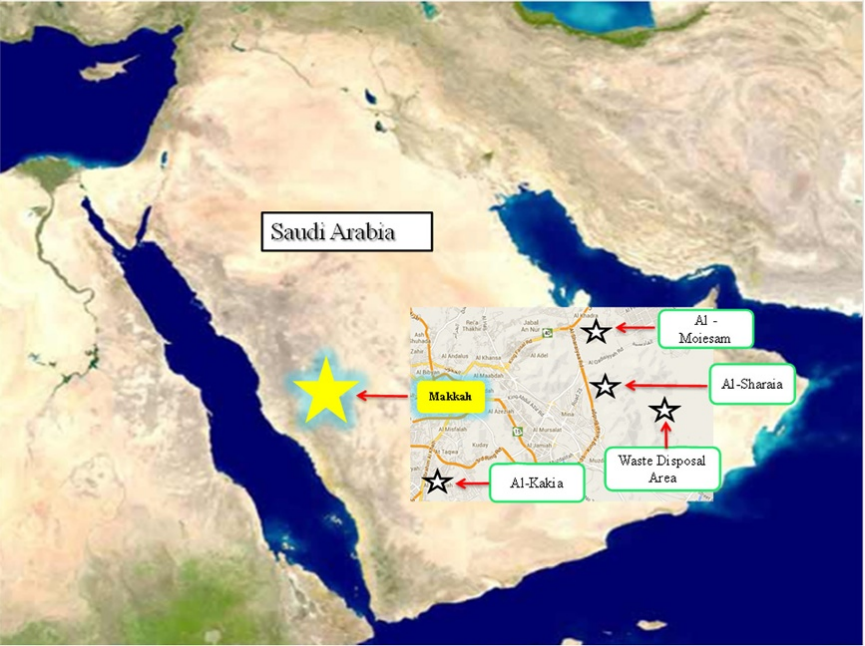
**Areas in Makkah whereas air samples were collected.** The map was from Wikipedia http://fr.wikipedia.org/wiki/Portail:Afrique.

Air samples were collected for three days (27–29 of October 2012 / 10–12 of DhuI Hijjah 1433) from four sites: slaughterhouses at Al-Kakia, Al-Meaisim and Al-Sharaia, and a waste disposable area specified for the remnants of slaughter (Figure 2). For each air sample, a volume of 1000 L was collected with a FCC-IV biological air sampler (AES Laboratory, France) mounted with a nutrient agar plate containing the antifungal agent amphotericin (Majed Al-Buqami Co. BMC, Saudi Arabia) according to the manufacturer’s instructions. Samples were collected on the first day from 7 am-9 am and from 7 pm-9 pm. On the second and third days, samples were collected from 10 am-3 pm. Weather parameters such as temperature and relative humidity were also recorded.

### Isolation of bacteria

Air sampled nutrient agar plates were sealed with parafilm and transported on the same day to the bacteriology laboratory at Special Infectious Agents Unit (SIAU) in King Fahd Medical Research Center and incubated at 37°C for 48 h. On the basis of macroscopic colony morphology, purification by sub-culturing for another 48 h at 37°C on the same media was done to obtain pure culture isolates. The selection of the colonies for further analysis was based on their size, shape, opacity, and texture. Stocks of purified strains were stored in 20% glycerol in cryogenic vials at -80°C and then transferred to Marseille at -80°C on dry ice in sterile conditions.

### MALDI-TOF mass spectrometry

**Figure 3:**
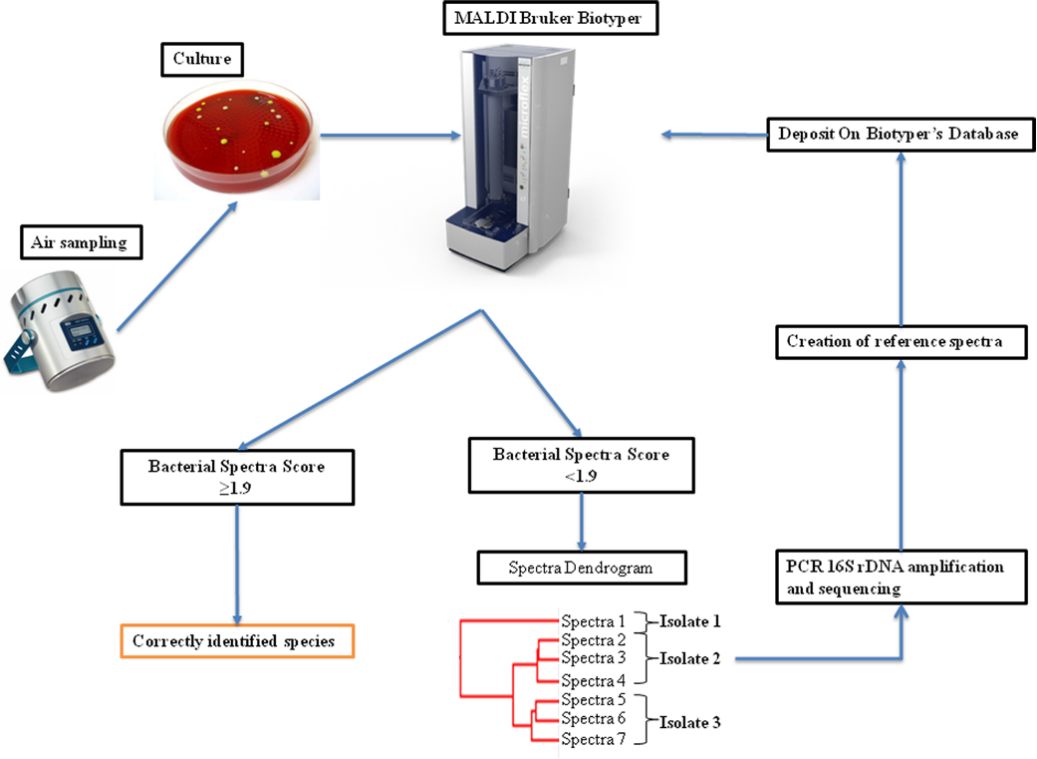
**Strategy used for the identification of isolates.**

We used a MALDI-TOF MS (Bruker Daltonics, Billerica, Mass., U.S.A.) to quickly identify bacterial colonies (Figure 3). Each isolate was deposited onto a MALDI-TOF target in two spots and each colony was covered with 2 ml of matrix solution (saturated α-cyano-4-hydroxycinnamic acid in 50% acetonitrile and 2.5% trifluoroacetic acid) without other supplements. The bacterial spectra were automatically acquired using flexControl 3.0 software, and the analysis was carried out with Biotyper 2.0 software. As previous described, a species was considered to be correctly identified when at least one spectrum presented a score ≥1.9 [14, 24]. For isolates with a score <1.9, a dendrogram was made from their spectra by the MALDI Biotyper 3.0 software. Unidentified isolates that clustered together were categorized as belonging to the same bacterial species, and only one isolate per cluster was used for PCR amplification and sequencing of the 16S rRNA gene. All of the bacterial spectra identified by 16S rRNA sequencing were added to the MALDI-TOF MS database, and we then performed a new scoring of the clustered species to verify that they were identical and to determine whether their matching score was ≥1.9. For isolates that did not cluster with other bacteria, PCR amplification and sequencing of the 16S rRNA gene was independently performed.

### Molecular assays

Total genomic DNA was extracted from isolates using a QIAamp tissue kit (Qiagen, Hilden, Germany). The genomic DNA was stored at 4°C until used for PCR amplification and sequencing of the 16S rRNA gene using the methods previously described [25]. All the sequences were compared with those available in the GenBank, EMBL, and DJB databases using the gapped BLASTN 2.0.5 program through the National Center for Biotechnology Information. A threshold similarity of >98.7% was used to define a new bacterial species [12].

### Statistical analysis

For the comparison of the different species isolated from the four different sites, the Student’s t-test or ×2 test was performed using EpiInfo version 6.0 software (Centers for Disease Control and Prevention, Atlanta, GA, USA). A *p* value < 0.05 was considered significant.

### Ethic statement

Our study design conformed to directives concerning the conduct of trials for Saudi Arabia. Ethic approval by the local ethic committee was not necessary.
